# Use of and Self-Perceived Need for Assistive Devices in Individuals with Disabilities in Taiwan

**DOI:** 10.1371/journal.pone.0152707

**Published:** 2016-03-29

**Authors:** Kwok-Tak Yeung, Chung-Hui Lin, Ya-Ling Teng, Fen-Fen Chen, Shu-Zon Lou, Chiung-Ling Chen

**Affiliations:** 1 Department of Occupational Therapy, Chung Shan Medical University, Taichung, Taiwan; 2 Occupational Therapy Room, Chung Shan Medical University Hospital, Taichung, Taiwan; University of Perugia, ITALY

## Abstract

Assistive devices (ADs) can help individuals with disabilities achieve greater independence, and it can enhance the quality of their lives. This study investigated the use of and self-perceived need for ADs in individuals with disabilities, and determined the influence of gender, age as well as type and degree of disability on the use of and self-perceived need for ADs. This descriptive study utilized a cross-sectional survey design with a convenience sample of participants. A total of 1018 subjects with disabilities who visited an exhibition of assistive technology and two ADs research and development centers completed a questionnaires either by themselves or via a caregiver who completed the questionnaire on behalf of the subject or via interviewers trained specifically for this study. The Mann-Whitney U test and Kruskal-Wallis test were used to determine the influence of participant characteristics on the use of ADs. The results showed that 77.2% and 83.3% of the participants reported that they used and needed AD(s) to engage in activities of daily living. The mean quantity of the use of and self-perceived need for total types of ADs were 3.0 and 5.3, respectively. Participants with different disabilities reported different percentages of the use of various types of ADs. No difference was found between genders and among the age groups in the use of quantity of ADs. Individuals with different types and degrees of disability used different quantities of ADs. Participants with physical, visual and multiple disabilities used significantly more ADs compared to participants with intellectual disability. The total quantity of ADs used increased significantly with increased severity of disability. The mean use of assistive devices was lower compared to the mean need of individuals with disabilities. Further study is required to determine why patients feel the need for but not currently use a specific assistive device.

## Introduction

The International Classification of Functioning, Disability and Health (ICF) describes human functioning in terms of body structures, body functions, activities, and participation. It states that disability is a condition that is influenced by the interaction between persons with health conditions and their environment [1]. Assistive devices (ADs) can help individuals with disabilities compensate for lost functions, increase their independence, develop their potential, and thus enhance their quality of life. Assistive technology (AT) has been commonly used in developed countries to help individuals with disabilities overcome obstacles when seeking medical treatment, pursuing education, obtaining employment or caring for families, and obtaining equal opportunities to participate in society. The Assistive Technology Act (Public Law 108–364), otherwise known as the Tech Act, was first passed by American Congress in 1988 as the Technology-Related Assistance for Individuals with Disabilities Act (Public Law 100–407) and has been reauthorized in 1994, 1998, and 2004. The Tech Act required States to provide individuals with disabilities with the needed AT and services [2]. Beginning in 1975, the Individuals with Disabilities Education Act (Public Law 108–446) was passed that defined both AT devices and services for children with disabilities for inclusion in the individualized family service plan and individualized education program [3]. The Standard Rules on the Equalization of Opportunities for Persons with Disabilities adopted by the United Nations in 1993 stated that the government should ensure the development and provision of supportive services, including ADs for persons with disabilities, in order to increase their daily independence and safeguard their rights [4].

The ICF and the laws made it clear that assistive technology included more than devices, but also user-driven and user-focused services. A number of assessment tools and models have been developed to guide AT service delivery such as the Student Environment Task Tool framework [5], the Matching Person and Technology model [6], the Human Activity Assistive Technology model [7], and the Assistive Technology Assessment model [8]. Each of these models acknowledges the impact of the environmental and personal factors in intervention, placing emphasis on a user-driven process and person-centered approach for AT service delivery.

In Taiwan, 1,141,677 people, or 4.87% of the total population, received the Disability Identification by the end of 2014 [9]. The statistical analysis of recent 10 years revealed an annual increase of the disabled population of approximately 2.32%, indicating that a growing number of disabled individuals need assistance to overcome obstacles, so welfare services for the disabled have become one of the major topics of government policy. In recent years, the government of Taiwan has paid much attention to the development and popularization of AT, and the rights of the disabled to the use of AT have been safeguarded through new legislation. The Physically and Mentally Disabled Citizens Protection Act of 1997, which has been amended and promulgated to the People with Disabilities Rights Protection Act in 2007, stipulates regulations concerning research and development of ADs related to health and medical care, education, employment, daily living and social participation [10]. As a result, various organizations have already provided assistance and financial support for the establishment of a number of ADs research and development centers.

The objectives of the establishment of ADs research and development centers are to develop devices and technologies for individuals with disabilities, and to provide expert evaluation and recommendation as well as individualized services. Outcomes of AT use are the result of a service delivery. It is important to undertake research that supports service delivery practices. Previous studies have shown that, to be effective, assistive devices and services should match with the user’s needs [11–15]. Research have also revealed that failure to take into consideration the user’s needs during AT evaluation is a primary reason for abandonment [13, 16–18]. To improve AT services and users’ functions and participation, it is essential to gain an understanding of user subjective needs and preferences. The gaps between current conditions or use conditions and desired conditions or self-perceived need conditions must be determined to appropriately identify the need.

However, studies on ADs use and need in Taiwan are scarce. In the report on physically and mentally disabled citizens living demand survey, Republic of China, 2000 [19], indicated that approximately 40% of all physically and mentally disabled citizens used various kinds of Ads. The follow-up surveys reported the similar conditions, 37.85% in 2003 [20], 35.76% in 2006 [21], and 38.77% in 2011 [22]. In a national survey of the usage of assistive devices, samples were limited to subjects who were more than 50 years of age with long-term disability [23]. Hu conducted a survey on the current usage conditions of locomotive assistive devices for long-term care clients [24]. All these surveys reported the usage conditions but not the needed conditions of ADs. Zeng and Li investigated the demand for daily living ADs in elderly, but interviewed only ten physically and mentally disabled senior citizens, making it impossible to obtain a complete picture of the demand for ADs [25]. Only one study was conducted to investigate the need of individualized assisted device in people with disabilities. However, the questionnaires were sent by post, only 539 out of 2850 questionnaires were returned, the return rate was rather low (18.9%) [26].

The objective of this study was to investigate the use of and self-perceived need for various categories of ADs by individuals with disabilities and to study whether gender, age, as well as type and degree of disability have any influence on the total quantity of ADs used. The aims are to bridge the gap between what people use and what people want to participate in daily living activities, and to provide research and development centers with a basis for program development and relevant government authorities with a policy reference.

## Methods

### Ethics statement

This study has been reviewed by the Chung Shan Medical University Hospital Institutional Review Board. The Board has determined that this study is exempt from review and approval is not required because it is an anonymous survey. A signed informed consent form was obtained from all participants after clearly introducing the survey.

### Participants

In this study, we used convenience sampling and interviewed individuals with disabilities who visited an exhibition of AT. Because the AT exhibition was held in northern area of Taiwan, most visitors with disability came from northern or central area of Taiwan. To consider samples that might vary by region, additional subjects were recruited from two ADs research and development centers, one in the southern and the other in the eastern area of Taiwan.

### Data collection

The data was collected using a self-reported “Use of and Need for Assistive Devices in Individuals with Disabilities” questionnaire comprising three sections: 1. Participants’ basic information, 2. Participants’ basic daily living abilities, and 3. ADs use and need. To establish content and face validity, five senior therapists who providing AT-related services examined the questionnaire. The senior therapists confirm that the questionnaire had acceptable face validity although some comments were given. Most comments about content validity were directed towards the questionnaire wording and adding some items of ADs in each category for participants to check. Three items were added, and five questions were rewritten or had words changes for clarity and understanding. Inter-rater reliability was not formally tested. For it is a closed-ended questionnaire that collects quantitative data, by using low inference descriptors that readily quantified, it is safe to say that the internal reliability of the questionnaire can be ensured.

Section 1 (participants’ basic information) included gender, age, living area, educational background, type of disability, and degree of disability. Section 2 (basic daily living abilities) collected the information about the participants’ level of function in basic activities of daily living (ADLs). The basic ADLs included eating, dressing, grooming, bathing, toileting, transferring, walking, and communication. Four levels of response categories were (a) able to perform the activity without using ADs, (b) able to perform the activity with occasionally use of ADs, (c) able to perform the activity with frequently use of ADs, and (d) unable to perform the activity requiring help from others. Section 3 (ADs use and need) collected the participants’ use of and self-perceived need for various categories of ADs. ADs were classified into ten categories according to function: eating, dressing, grooming/bathing, toileting, transferring, walking, communication, rehabilitation, computer usage, and reading/learning, and in each category, the participants were asked to check which AD they currently used and which AD they may needed. Participants could select “none” and indicate a number of AD items that they used and needed. To assure consistency between sections 2 and 3, we checked whether the respondents had not forgotten to complete section 3 (ADs use and need). For example, if under the activity “dressing” a respondent had checked “able to perform the activity with frequently use of ADs”, we checked whether the category “dressing ADs” was also checked under ADs use and need in section 3.

The questionnaires were completed either by self-report, by a caregiver on behalf of the participants, or by interviewers trained specifically for this study. The choice of administration format depended on the characteristics of the participants (if they were able to read and write), and it was determined by the interviewers. The interviewers conducted interviews or facilitated the administration process, answered any question, and checked for missing data when the questionnaires were self-administrated or completed by a caregiver on behalf of the participant.

### Data analysis

Descriptive statistics, including frequency distribution and percentages, were used to describe the participants’ basic information, abilities in basic ADLs, and the use of and need for ADs. In section 3, one AD item checked in each category being considered as one device, the number of items (devices) checked in each category were added up to represent the total number of ADs used and needed by participants. The mean and standard deviation of total quantity of ADs the participants used and needed were calculated. Since the data were not normally distributed, the Mann-Whitney U-test and Kruskal-Wallis one-way analysis of variance were used to determine any differences in the total quantity of ADs used by different participant characteristics (gender, age, type of disability, degree of disability). To minimize type I error due to multiple testing, the significance level was set at α = 0.01.

## Results

A total of 1018 questionnaires were completed, of which 845 were collected in the AT exhibition, the other 163 were collected from the ADs research and development centers. Ninety-nine questionnaires were collected from a center in the southern area and 74 questionnaires were collected from a center in the eastern area.

### Basic information

Participants comprised 588 males (57.8%) and 430 females (42.2%). Basic participants’ characteristics are outlined in Table 1. Regarding age distribution, most participants (259or 25.4%) were younger than 20 years of age, while the proportion of participants older than 20 years of age decreased as a function of age. The largest number of participants, namely 564 or 55.4%, had a physical disability, and the highest proportion of participants (427 or 41.9%) had a severe disability (Table 1). In comparison with the target population, significant differences were found between types of disability (X^2^ = 126.5, p<0.0001) and severity of disability (X^2^ = 465.1, p<0.0001). There was no significant difference between gender (X^2^ = 0.16, p = 0.69).

**Table 1:** Basic demographic characteristics of the participants.

Demographic characteristics	Number	Percent
Total	1018	100
Gender		
Male	588	57.8
Female	430	42.2
Age (years)		
<20	259	25.4
20–29	222	21.8
30–39	190	18.7
40–49	184	18.1
≥ 50	163	16.0
Area of living		
Northern	585	57.5
Central	152	14.9
Southern	169	16.6
Eastern	112	11.0
Level of education		
Non-high school graduate	384	37.7
High-school graduate	430	42.2
College graduate	204	20.1
Types of disability		
Physical disability	564	55.4
Intellectual disability	97	9.5
Visual disability	31	3.0
Hearing disability	38	3.7
Speech/language disability	13	1.3
Autism	16	1.6
Multiple disability	237	23.3
Miscellaneous	22	2.2
Severity of disability		
Mild	143	14.0
Moderate	333	32.7
Severe	427	41.9
Profound	115	11.7

### Abilities in basic activities of daily living

As shown in Table 2, a higher percentage of participants were able to perform feeding (71.7%), communication (69.7%) and upper extremities dressing (68.9%) activities without ADs. Walking and bathing were the activities that a relatively high percentage of participants were able to perform with the occasional or frequent use of ADs while bathing, toileting and lower extremities dressing were the activities that a higher percentage of participants were unable to perform and for which help from others was required.

**Table 2:** Number and percentage of participants in four levels of function in various types of basic ADLs.

ADLs	Able to perform Without ADs		Able to perform Occasionally use ADs		Able to perform Frequently use ADs		Unable to perform	
	N	%	N	%	N	%	N	%
Feeding	730	71.7	118	11.6	50	4.9	120	11.8
UE dressing	701	68.9	58	5.7	40	3.9	219	21.5
LE dressing	663	65.1	85	8.3	43	4.2	227	22.3
Grooming	685	67.3	93	9.1	103	10.1	137	13.5
Bathing	500	49.1	140	13.8	118	11.6	260	25.5
Toileting	563	55.3	109	10.7	114	11.2	232	22.8
Transferring	650	63.9	68	6.7	81	8.0	219	21.5
Walking	358	35.2	189	18.6	365	35.9	106	10.4
Communication	710	69.7	84	8.3	58	5.7	166	16.3

ADLs: activities of daily living, N: number, ADs: assistive devices, UE: upper extremities, LE: lower extremities

### Assistive devices use and self-perceived need

Table 3 shows the percentage of participants who use of and need of ADs across the ten categories of ADs. It also lists three AD items used and needed by the highest percentage of participant across each category of ADs. The mean percentage of use and need for all categories of ADs were 22.2% and 32.9%, respectively. Most participants reported the greatest use of and need for ADs for walking (54.5% and 56.8% respectively) and rehabilitation (39.8% and 49.8% respectively).

**Table 3:** The percentage of participants who used and needed ADs as well as three AD items with the highest percentage of participants who used and needed in each type of ADs.

Category of ADs	Used(%)	Category of ADs	Needed(%)
AD Items		AD Items	
**Feeding**	16.2	**Feeding**	22.8
Lap board	5.2	Special utensils	8.6
Special utensils	4.4	Lap board	7.1
ADs for grasp	1.7	Special plate/bowl	7.0
**Dressing**	12.9	**Dressing**	23.0
ADs for shoes wearing	4.2	ADs for shoes wearing	9.2
ADs for LE dressing	1.7	ADs for LE dressing	7.9
Special clothes	1.7	ADs for UE dressing	6.4
**Grooming/Bathing**	25.4	**Grooming/Bathing**	42.0
Bath bench	12.0	Bath bench	21.5
Anti-slip mats	7.6	Anti-slip mats	17.7
Lever handle faucet	5.6	Shower chair	8.6
**Toileting**	22.0	**Toileting**	28.9
Commode chair	8.0	Commode chair	12.8
Bed pans and urinals	5.5	Raised toilet seat	7.0
Urine condoms	3.3	Bed pans and urinals	6.3
**Transferring**	14.7	**Transferring**	28.6
Bed rails	6.4	Bed rails	11.6
Lift system	2.1	Lift system	7.2
Ceiling hoist	1.8	Floor hoist	6.1
**Walking**	54.5	**Walking**	56.8
Wheelchair	26.6	Wheelchair	26.6
Walking aids	21.4	Walking aids	19.2
LE orthoses	12.6	Powered wheelchair	15.8
**Communication**	13.7	**Communication**	27.5
Communication board	5.3	Communication board	15.2
Hearing aid	3.7	ADs for telephone	6.1
ADs for telephone	2.4	Hearing aid	5.0
**Rehabilitation**	39.8	**Rehabilitation**	49.8
LE orthoses	11.6	Cushion	15.9
Cushion	11.4	Standing frame	13.0
Special shoes	9.3	LE orthoses	12.3
**Computer usage**	8.6	**Computer usage**	20.2
Special mouse	3.8	Special mouse	9.4
Typing stick	1.5	Typing stick	4.3
Special key board	1.4	Special key board	8.9
**Reading/Learning**	13.9	**Reading/Learning**	30.1
Magnification device	5.0	Communication board	12.1
Communication board	4.7	ADs for writing	9.0
Hearing aid	3.0	Magnification device	7.6

### Influence of the characteristics on the use of and need for ADs

For each type of disability, the percentage of participants that used a particular category of AD is shown in Table 4. Participants with physical disability and multiple disabilities used mostly rehabilitation ADs (52.5% and 46.5%, respectively) and walking ADs (75.6% and 44.9%, respectively). Participants with intellectual disability were least likely to use ADs; however, in this group the use of communication aids (14.3%) and reading/learning aids (19.4%) was higher compared to the use of other categories of ADs. Participants with visual disability used mostly reading/learning ADs (71.0%), followed by walking ADs (58.1%). Participants with a hearing disability used primarily communication ADs (71.1%), followed by reading/learning ADs (55.3%). Autistic participants used mostly reading/learning ADs (43.8%) and communication ADs (37.5%). Only 13 participants had a language disability, and used relatively few ADs.

**Table 4:** Number of participants in each type of disability and percentage of which used various categories of assistive devices.

Assistive Devices	Types of Disability							
	Number							
	Physical	Intellectual	Visual	Hearing	Speech Language	Autism	Multiple	Miscellaneous
	564	97	31	38	13	16	237	22
Feeding	14.7	8.2	25.8	2.6	0	25	25.5	0
Dressing	13.6	5.1	6.2	2.6	0	12.5	16.5	9.5
Grooming/Bathing	30.0	13.3	19.4	7.9	7.7	31.3	24.7	9.5
Toileting	24.6	3.1	9.7	5.3	7.7	18.8	29.6	14.3
Transferring	17.3	5.1	19.4	5.3	7.7	12.5	14.0	14.3
Walking	75.6	5.1	58.1	7.9	15.4	12.5	44.9	23.8
Communication	5.5	14.3	25.8	71.1	15.4	37.5	22.6	19.0
Rehabilitation	52.5	7.1	16.1	7.9	15.4	12.5	46.5	23.8
Computer usage	8.0	4.1	22.6	5.3	7.7	31.3	10.3	9.5
Reading/Learning	8.5	19.4	71.0	55.3	15.4	43.8	21.4	4.8

Overall, 232 (22.8%) and 170 (16.7%) did not use or need any ADs. The percentages of the participants who used and needed at least one AD for their daily living were 77.2% and 83.3%, respectively. The mean use of the total number of AD items was 3.0 (±3.0) while the mean self-perceived need for the total number of AD items was 5.3 (±5.7). The differences between males and females or by age groups in the use of total quantity of ADs were non-significant (U = 117184.5, p>0.01). A significant difference was found in the use of total quantity of ADs by type of disability (X^2^ = 122.1, p<0.001) and degree of disability (X^2^ = 110.9, p<0.001). Post-hoc tests revealed that participants with physical, visual, or multiple disabilities used significantly more ADs compared to participants with intellectual disability (U = 1072.0, U = 603.5, U = 5108.5, respectively, all p<0.01). The total quantity of ADs used increased significantly as a function of severity (mild-moderate U = 16989.5, mild-severe U = 14976.5, mild-profound U = 4533.5, moderate-severe U = 50307.5, moderate-profound U = 15260.5, all p<0.01) while the severe and profound groups did not differ significantly from one another.

## Discussion

Comparing sample demographic data with the target population, there were significant differences between types of disability and severity of disability but there was no significant difference between gender. This points to a fault in the convenience sampling and that will be discussed later. Comparison of age, area of living, and level of education were not performed because the category that this study used was different from the disabled population database [9].

In the report on physically and mentally disabled citizens living and demand assessment survey, Republic of China, 2011, indicated that approximately 40% of all physically and mentally disabled citizens needed and used various kinds of ADs [22]. Compared to the survey results in 2000 [19], 2003 [20], and 2006 [21], the ratio of the needs for ADs in individuals with disabilities was almost the same. The percentages for the use and need (77.2% and 83.3% respectively) were higher in our study because the participants recruited from an exhibition of AT and ADs research and development centers should have already used some kind of ADs or have greater need for ADs.

When comparing sample characteristics of this study to the statistical data of the Republic of China report, 2011 [22], the study conducted by Leong et al. [26], and the Tainan [27] report on living demand in physically and mentally disabled citizens, in which the ratio of male to female subjects was approximately 6 to 4 (57.24%:42.76% 60.8%:39.2%, and 61.1%:38.5%, respectively), we see that the male-female ratio in this study was similar, specifically, 57.8% to 42.2%. Concerning age, most participants in Leong’s study were 20–40 years old and in the Tainan report were 25–44 years old, which is similar to the current study in which most subjects were 20–49 years old. The highest percentage of subjects in the Republic of China report, however, were >65 years old (34.3%) while this study had only a small number of participants older than 50. This may be explained by the fact that the participants in this study were from an AT exhibition and two ADs research and development centers. Older individuals may not be so active to seek help from external resources. In Leong’s study, the highest proportion of participants had a physical disability (76%), followed by multiple disabilities (9%), hearing disabilities (9%), and intellectual disabilities (6%). According to physically and mentally disabled population data of the Ministry of Health and Welfare [9], the largest number of people had a physical disability (33.72%) while multiple disabilities and intellectual disability accounted for 10.38% and 8.84%, respectively. The Republic of China report also showed the highest percentage of people with a physical disability (35.58%), followed by hearing, multiple, and intellectual disabilities. In our study, the highest percentage of subjects had a physical disability (55.4%), followed by multiple, intellectual, and hearing disabilities, but the percentage of multiple disabilities (23.3%) was higher compared to that in the Republic of China report.

Individuals with disabilities in this study had the greatest need for walking, rehabilitation, and grooming/bathing aids, which is consistent with the findings of a study conducted with elderly as subjects. In their study on community elderly, Edwards and Jones [28] indicated that people older than 75 years of age showed increased use of walking aids and wheelchairs and most commonly used walking and grooming/bathing aids, such as a bath mat, walking stick, and bathroom handrails. Study by Sonn et al. also showed that urban elderly commonly use hygiene and mobility devices, with 24% of subjects under 80 and 57% of subjects above 80 years of age using such ADs [29]. George and colleagues studied ADs use among home-based elderly over 75 years of age, and found that many elderly with function loss needed ADs for bathing and toileting [30]. In Taiwan, in a nation-wide usage survey of assistive device in long-term disabled subjects who were more than 50 years of age, locomotive devices were found to be the most used type of Ads (82%) [23]. A study conducted with stroke patients in Taiwan also indicated that more than 90% of stroke patients used ADs. The most commonly used types of ADs were walking aids (76.9%) and bathing aids (10.6%) [31]. The findings of this study were also consistent with the findings of Leong et al. [26].

In this study, we demonstrated that participants with a different type of disability used different types of ADs and participants with physical, visual or multiple disabilities used more ADs compared to participants with intellectual disability. The previous study also noted that people with intellectual disabilities underutilize assistive technology aids [32]. The existing barriers to the use of ADs include lack of information about the availability and cost of the ADs as well as limited selection of ADs, among others [33]. In the literature, most studies on ADs have focused on one specific diagnosis/disability group or one specific category of ADs. For example, Mannet al. studied AD use among home-based elderly with arthritis indicating that elderly with arthritis use multiple ADs (10 items on average) [34]. Additionally, Holme et al. studied the advantages of the use of environmental control units in patients with spinal cord injury [35]. Another study by Haworth et al. investigated the use of aids by hip replacement patients [36] while Mann et al. compared the AD use and needs in home-based elderly with different impairments [37]. Some other studies have focused on the use of bath aids in multi-diagnostic samples [38], the use of alternative communication systems in people with severe motor impairment [39], adaptive seating for wheeled mobility devices used by children and youth with wheeled mobility needs [40], the usage conditions of locomotive assistive devices for long term care clients [24], and the status in computer access and the use of related assistive devices in students with physical disabilities [41]. Future studies should therefore focus on the use of one type of ADs or on subjects with one specific disease or type of disability.

There was no difference between the genders in the use of total quantity of ADs in this study. Study by Edwards and Jones; however, indicated that the use of certain ADs in older people differs by gender. They found that female subjects used more walking and bathing aids compared to male subjects [28]. A survey conducted in the USA with adult US resident using wheeled mobility equipment in 2005 found that wheeled mobility equipment users are more likely to be older, female, and in poor health compared to the general population [42]. Study on factors that influence the use of ADs in disabled community elderly showed a correlation between the use of ADs and social isolation of women, low education, and residence in rural areas [43]. The participants in our study were generally younger compared to those in the above-mentioned studies; hence the gender effects cannot be determined.

The degrees of disability influenced the total quantity of ADs utilized. This result was similar to the findings obtained by Verbrugge et al. [44], who found that severe disability and poor overall health/disability status increased the use of ADs. Thyberg et al. [45] also suggested that severe disease and greater disability are associated with greater use of ADs. However, the results in our study did not show any differences between severe and profound degree of disability. We supposed that in addition to use of ADs, the participants with profound degree of disability receive much more help from others. A prior study has also shown that high numbers of ADLs, instrumental ADLs, and physical limitations are strongly related to increased use of personal and equipment compared with equipment only [44]. This finding is not surprising, given the evidence from this study, the aforementioned studies, and one similar to it [26]. It indicated that AT professionals should endeavor to expand their knowledge and expertise to provide services in proportion to the severity of disability. Further study with special emphasis on persons with more severe disabilities is required to include a better assessment of the severity of the disability and a better classification of ADs to obtain a better understanding of how much persons with disabilities and their families are able to invest in AT on their own and what share of the cost the public should bear.

A relatively high percentage of participants were unable to perform basic ADLs such as dressing, bathing, toileting, and transferring, and required help from others. The previous surveys in the literature have consistently demonstrated a trend toward an increased AD use and decreased use of personal assistance by older people with varying levels of functional disability and impairment [43, 46, 47]. Future study should investigate whether increased use of ADs can reduce the need for personal care in individuals with profound disability.

The use of ADs quantity was lower compared to the self-perceived need for ADs quantity. These findings confirmed that there is a gap between objectively- or professionally-assessed and subjectively- or self-perceived need. Therefore, for beneficial outcomes to be experienced, users have to be party to decisions about the provision of AT. These findings also give support to the relevance of user-driven process and person-centered approach for a successful AT assessment and delivery. Further study is required to determine whether the gap is the result of the lack of understanding of ADs, the difficulty of obtaining ADs, the lack of suitable ADs, the lack of service delivery, high prices, the lack of subsidy funding, or the lack of centralized information and evaluation systems, among others [48].

One limitation of the current study was that the non-probability convenience sample may limit the generalizability of this study. Restricted by budget and manpower, we used convenience sampling of a certain group of individuals with disabilities, instead of sampling of the entire population. However, the sample size was considerably large. The individuals with disabilities from an AT exhibition and two ADs research and development centers have a relatively better understanding of, care more for, are interested in, and feel the need for ADs. Questionnaires were collected in person from individuals with disabilities or their caregivers, so the response rate was rather high. Only a small number refused to be interviewed. By promptly answering any questions that participants had, the response accuracy increased. The results of this study are therefore of considerable referential value. Another limitation was that this study did not explore why participants feel they need but do not currently use a specific AD.

Future research could be improved by using a stratified sampling where the sample should be further divided into groups according to gender, age and type of disability as is done in the database of physically and mentally disabled citizens of the Ministry of Health and Welfare [9]. Further study is required to find out why patients feel they need but do not currently use a specific AD. Future study may also probe into patients’ use of AD, including the frequency of and satisfaction with the use of these devices, as well as into the influence of different cultural backgrounds on AD use and need.

## Conclusions

The questionnaire data were collected using a large sample and face-to-face interviewing; thus the results of this study are therefore of considerable referential value. The highest percentage of subjects had a physical disability and the highest usage percentage was found for walking, rehabilitation and grooming/bathing while computer access, dressing, and communication aids showed the lowest usage percentage. No difference was found between the genders and among the age groups in the use of quantity of ADs while type and degree of disability influenced the number of ADs used. These findings support that conducting additional research to improve knowledge of assisted device usages for this subject population should be given high priority. Subjects with a different type of disability needed different types of ADs. The mean quantity of the use of and the mean self-perceived need for total types of ADs were 3.0 and 5.3, respectively. This study confirms that there is a gap between use conditions and self-perceived need conditions. It gives support to the thesis that effective AT service delivery should place emphasis on a user-driven process and person-centered approach. It is hoped that the current study may provide valuable information for clinicians to better understand the needs of the users of AT, for organizations to have a basis for program development, and for policymakers to consider in future policy making.
